# Barium Titanate (BaTiO_3_) Nanoparticles Exert Cytotoxicity through Oxidative Stress in Human Lung Carcinoma (A549) Cells

**DOI:** 10.3390/nano10112309

**Published:** 2020-11-22

**Authors:** Maqusood Ahamed, Mohd Javed Akhtar, M.A. Majeed Khan, Hisham A. Alhadlaq, Aws Alshamsan

**Affiliations:** 1 King Abdullah Institute for Nanotechnology, King Saud University, Riyadh 11451, Saudi Arabia; mjakhtar@ksu.edu.sa (M.J.A.); mmkhan@ksu.edu.sa (M.A.M.K.); hhadlaq@ksu.edu.sa (H.A.A.); 2 Department of Physics and Astronomy, College of Science, King Saud University, Riyadh 11451, Saudi Arabia; 3 Department of Pharmaceutics, College of Pharmacy, King Saud University, Riyadh 11451, Saudi Arabia; aalshamsan@ksu.edu.sa

**Keywords:** BaTiO_3_ nanoparticles, selective cytotoxicity, oxidative stress, antioxidant enzymes, cancer therapy

## Abstract

Barium titanate (BaTiO_3_) nanoparticles (BT NPs) have shown exceptional characteristics such as high dielectric constant and suitable ferro-, piezo-, and pyro-electric properties. Thus, BT NPs have shown potential to be applied in various fields including electro-optical devices and biomedicine. However, very limited knowledge is available on the interaction of BT NPs with human cells. This work was planned to study the interaction of BT NPs with human lung carcinoma (A549) cells. Results showed that BT NPs decreased cell viability in a dose- and time-dependent manner. Depletion of mitochondrial membrane potential and induction of caspase-3 and -9 enzyme activity were also observed following BT NP exposure. BT NPs further induced oxidative stress indicated by induction of pro-oxidants (reactive oxygen species and hydrogen peroxide) and reduction of antioxidants (glutathione and several antioxidant enzymes). Moreover, BT NP-induced cytotoxicity and oxidative stress were effectively abrogated by N-acetyl-cysteine (an ROS scavenger), suggesting that BT NP-induced cytotoxicity was mediated through oxidative stress. Intriguingly, the underlying mechanism of cytotoxicity of BT NPs was similar to the mode of action of ZnO NPs. At the end, we found that BT NPs did not affect the non-cancerous human lung fibroblasts (IMR-90). Altogether, BT NPs selectively induced cytotoxicity in A549 cells via oxidative stress. This work warrants further research on selective cytotoxicity mechanisms of BT NPs in different types of cancer cells and their normal counterparts.

## 1. Introduction

Barium titanate (BaTiO_3_) nanoparticles (BT NPs), a perovskite-type ceramic material, has exceptional characteristics such as high dielectric constant and suitable ferro-, piezo-, and pyro-electric properties. BT NPs are broadly used in the manufacture of multilayer ceramic capacitor, thermistors, transducers, infrared detectors, sensors, and electro-optical devices [1,2]. Studies also suggested that BT NPs have potential to be applied in biomedical fields, including tissue engineering, drug delivery, and cancer therapy [3,4,5,6]. For instance, BT NPs enhance the cellular uptake of the anticancer drug doxorubicin [7]. BT NPs are also being investigated as bone repair material due to their excellent osteoinductivity [6,8]. Nevertheless, research on the underlying mechanisms of biological effects of BT NPs at cellular and molecular levels is scarce. Therefore, thorough study on the interaction of BT NPs with human cells is indispensable before their applications in biomedical field.

Investigation on underlying mechanisms of toxicity of NPs is still in progress. However, excessive production of reactive oxygen species (ROS) and oxidative damage of cell macromolecules have been established now as some of the key toxicity mechanisms of NPs. Oxidative stress happens when the intracellular level of ROS increases beyond a certain level and limits the antioxidant defense ability of cells. Several recent studies suggested that NPs induce cytotoxicity through ROS generation and oxidative damage of cellular constituents [9,10,11]. Recent research also indicated that generation of intracellular ROS levels in a controlled way can be exploited as a therapeutic tool for cancer treatment [12,13]. In this regard, relatively higher levels of ROS observed in cancer cells as compared to their normal counterparts represent a promising tool to target cancer cell selectively [12].

Studies on nanotechnology-based novel drug delivery and cancer therapy are now gaining recognition [14,15,16]. Several studies have demonstrated that defined dosages of semiconductor NPs potentially killed cancer cells, while not much affecting the non-cancerous normal cells [17,18,19]. For example, ZnO NPs have shown inherent properties of killing cancer cells while sparing the normal cells [20,21]. Our recent work indicated that SnO_2_ NPs have inherent characteristics of exerting cytotoxicity to human breast cancer cells while sparing the normal human lung fibroblasts (IMR-90) [22]. Metal doped TiO_2_ NPs also showed selective toxicity against cancer cells [19,23]. These semiconductor NPs may utilize the extreme conditions of oxidative stress of cancer cells. Increases in intracellular ROS to a certain level due to NP exposure may damage the already ROS-stressed cancer cells without much affecting the relatively less stressed normal cells.

This work was designed to investigate the cytotoxicity mechanisms of BT NPs in human lung carcinoma (A549) cells. The objective of the present study was conducted by estimating the cell viability, cellular morphology, activity of apoptotic enzymes (caspase-3 and -9), and mitochondrial membrane potential in A549 cells following exposure to BT NPs for 24 h. Cytotoxicity mechanisms of BT NPs were further delineated by measuring the several markers of oxidative stress, such as ROS, hydrogen peroxide (H_2_O_2_), and various antioxidant enzymes. Human lung cancer cell line (A549) was selected in this study because lung cancer is a leading cause of cancer-related mortality among men and women [24]. To check the selective cytotoxicity of BT NPs against A549 cancer cells, we also assessed the cytotoxic potential of BT NPs in normal human lung fibroblasts (IMR-90).

## 2. Materials and Methods

### 2.1. Synthesis of BaTiO_3_ Nanoparticles

Barium titanate (BaTiO_3_) nanoparticles were prepared by the facile sol–gel hydrothermal method using titanium tetrachloride (TiCl_4_) and barium chloride (BaCl_2_) as precursor materials. In brief, 1 mL of TiCl_4_ was diluted in 2 mL of HCl (2M) to prepare a slightly yellow solution. Then, 2.5 g of BaCl_2_ was dissolved in 20 mL of deionized water. The two solutions were mixed to form a barium titanium solution. Under stirring and N2 bubbling, 15 mL of NaOH (6M) was added to this solution to form a homogeneous colloidal barium titanium slurry. The colloidal solution of barium titanium was transferred into 50 mL Teflon-lined stainless autoclave and heated at 100 ^°^C for 2 h. After the completion of reaction, the autoclave was allowed to cool to room temperature. The prepared solid white powder of barium titanate was collected from the autoclave, washed several times with distilled water and ethanol, and dried at 60 ^°^C for h in a vacuum.

### 2.2. Characterization of BaTiO_3_ Nanoparticles

Crystallinity and phase purity of prepared BT nanopowder were examined by powder X-ray diffraction (PXRD) (Malvern, WR14 1XZ, UK) equipped with a PanAnalytic X’Pert Pro X-ray (Malvern, UK) diffractometer with nickel filter using Cu-Kα radiation (*λ* = 0.154 nm at 45 kV and 40 mA) as the X-ray source. BT NPs were also characterized by micro-Raman spectroscopy (Horiba Raman system, IY-Horiba-T64000) (Northampton, NN3 6FL, UK).

Surface morphology of BT nanopowder was assessed by field emission scanning electron microscopy (FESEM, JSM-7600F, JEOL, Inc., Tokyo, Japan) at an accelerating voltage of 15 kV. Shape and size of BT NPs were determined by field emission transmission electron microscopy (FETEM, JEM-2100F, JEOL, Inc., Tokyo, Japan) at an accelerating voltage of 200 kV. Briefly, a diluted suspension of BT NPs (50 µg/mL in ethanol) was sonicated in a sonicator bath for 10 min at 40 W to obtain a homogenous mixture. Then, a drop of suspension was placed onto a TEM grid, air dried, and observed with FETEM. 

Hydrodynamic size and zeta potential of BT NPs in distilled water and culture media were estimated by dynamic light scattering (DLS) (ZetaSizer Nano-HT, Malvern Instruments, UK). In brief, BT nanopowder was suspended in distilled water and culture medium at a concentration of 50 µg/mL. Then, the suspension was sonicated by a sonicator bath at room temperature for 10 min at 40 W, and DLS experiments were performed. We chose a 50 µg/mL concentration of BT NPs for DLS measurement, because this concentration was used in most of the biochemical studies.

### 2.3. Cell Culture and Exposure of BaTiO_3_ Nanoparticles

Human lung cancer cells (A549) and non-cancerous human lung fibroblasts (IMR-90) (ATCC, Manassas, VA, USA) were maintained in Dulbecco’s modified Eagle’s medium (DMEM, Invitrogen, Carlsbad, CA, USA) with the supplementation of 10% fetal bovine serum (FBS, Invitrogen), 100 U/mL penicillin, and 100 µg/mL streptomycin (Invitrogen) at 37 °C in a humidified 5% CO_2_ incubator.

To remove endotoxin contamination (if present), prepared NPs were autoclaved at 121 °C for 30 min using saturated steam under 15 pounds per square inch (psi) of pressure. An endpoint chromogenic limulus amebocyte lysate (LAL) assay further confirmed that prepared BT NPs were not contaminated from endotoxins (Figure S1 of Supplementary Materials).

Cells were exposed for 24, 48, or 72 h to different concentrations (5–200 µg/mL) of BT NPs. Briefly, BT NPs were suspended in culture medium (DMEM + 10% FBS) and diluted to desired concentrations (5–200 µg/mL). Different dilutions of NPs were further sonicated utilizing a sonicator bath at room temperature for 10 min to avoid agglomeration of NPs before exposure to cells. The ZnO NPs (25 µg/mL) was used as a positive control [22,25]. Preparation of ZnO NPs suspension was similar to BT NPs. In some experiments, cells were co-exposed with 2 mM of N-acetylcysteine (NAC) (Sigma-Aldrich, St. Louis, MO, USA) in the presence or absence of BT NPs/ZnO NPs. Cells without NP exposure served as controls in each experiment.

### 2.4. Cytotoxicity Study

Both MTT (3-(4,5-dimethylthiazol-2-yl)-2,5-diphenyltetrazolium bromide) [26] and NRU (neutral red uptake) [27] methods were used for the cell viability assay with some important changes [28]. Mitochondrial membrane potential was recorded with a microplate reader (Synergy-HT, BioTek, Winooski, VT, USA) and a fluorescent microscope (DMi8, Leica Microsystems, GmbH, Wetzlar, Germany) using the probe rhodamine-123 (Rh-123) (Sigma-Aldrich) [29]. Caspase-3 and caspase-9 enzyme activities were determined using colorimetric assay kits of BioVision kits. Morphologies of cells were captured through a phase-contrast microscope (DMIL, Leica Microsystems). Brief descriptions of each parameter of cytotoxicity are given in the Materials and Methods section of the Supplementary Materials.

### 2.5. Oxidative Stress Study

Intracellular levels of reactive oxygen species (ROS) were recorded with a microplate reader (Synergy-HT, BioTek, Vinooski, VT, USA) and a fluorescent microscope (DMi8, Leica Microsystems, Wetzlar, Germany) using the fluorescent probe 2’-,7’-dichlorodihydrofluorescein diacetate (H_2_DCFDA, Sigma-Aldrich) [29,30]. Intracellular hydrogen peroxide (H_2_O_2_) level was estimated by a MAK164 fluorescence kit (Sigma-Aldrich, St. Louis, MO, USA). Glutathione content was assayed using Ellman’s method [31]. The activity of glutathione reductase (GR) enzyme was assayed according to the procedure described by Carlberg and Mannervik [32]. Rotruck’s protocol was applied for the glutathione peroxidase (GPx) enzyme assay [33]. A colorimetric assay of the superoxide dismutase (SOD) enzyme was done using a kit from the Cayman Chemical Company (Michigan, OH, USA) [34]. Protein estimation was done by Bradford’s method [35]. Brief procedures of each parameter of oxidative stress are given in the Materials and Methods section of the Supplementary Materials.

### 2.6. Statistical Analysis

One-way analysis of variance (ANOVA) followed by Dunnett’s multiple comparison tests were used for statistical analysis. A *p*-value <0.05 was assigned as statistically significant.

## 3. Results and Discussion

### 3.1. Characterization of BaTiO_3_ Nanoparticles

**Figure 1:**
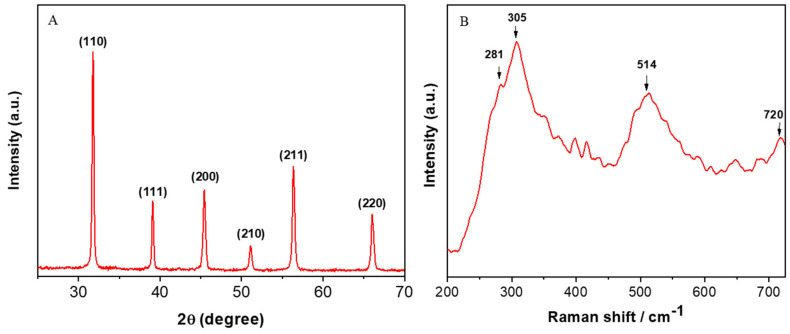
(**A**) XRD and (**B**) Raman spectra of barium titanate nanoparticles (BT NPs). XRD: X-ray diffraction.

Figure 1A represents the XRD spectra of BT NPs. All the peaks were well-matched with JCPDS no. 892475, exhibiting the formation of the cubic phase of BT NPs. The peaks at 31.81°, 39.12°, 45.44°, 51.08°, 56.34°, and 66.06 were attributed to (110), (111), (200), (210), (211), and (220) planes, respectively. Average particle size was determined from the strongest peak (110) using Scherrer’s equation [19]. The average particle size was estimated to be around 15 nm. Raman spectroscopy is a very sensitive technique to probe atomic structures of materials. Raman spectra of prepared BT NPs are given in Figure 1B. Peaks at 281 cm^−1^, 305 cm^−1^, 514 cm^−1^, and 720 cm^−1^ presented Raman shifts of BT NPs. These peaks suggested the crystalline cubic phase of BT NPs [36,37]. This result was in agreement with XRD spectra. 

**Figure 2:**
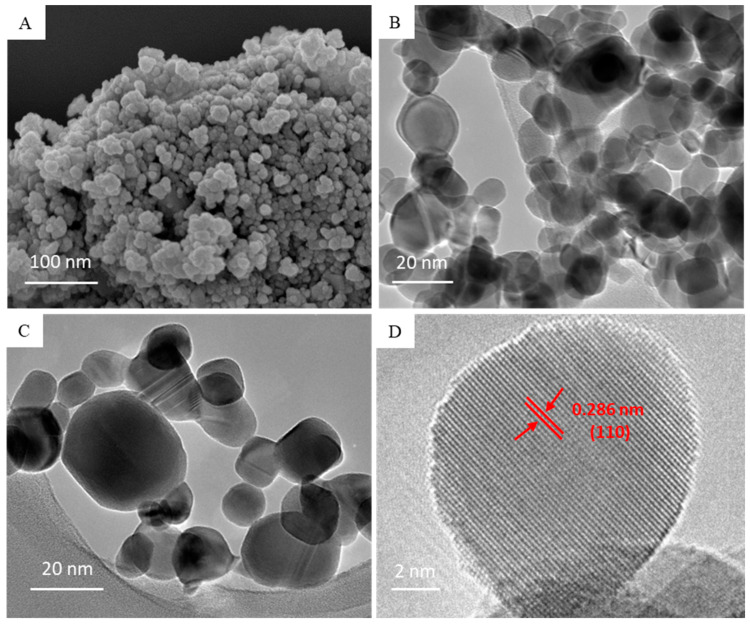
(**A**) FESEM, (**B** and **C**) low resolution FETEM, and (**D**) high resolution FETEM images of BT NPs. FESEM: field emission scanning electron microscopy, FETEM: field emission transmission electron microscopy.

Morphological characterization was done by FESEM and FETEM. Figure 2A–C represent the typical SEM and TEM images of BT NPs. The average size calculated from TEM image (>100 particles) was around 16 nm, which was well-matched with the size calculated from XRD spectra. A high resolution TEM image (Figure 2D) suggested the clear lattice fringes and crystallinity of BT NPs, supporting the XRD data. The distance between adjacent planes was found to be 0.286 nm, corresponding to the (110) plane of the cubic BT NPs (Figure 2D). Characterization data of BT NPs were in accordance with recently reported studies [6,38].

Physicochemical characterization of BT NPs in distilled water and culture medium is given in Table 1. Hydrodynamic size of BT NPs was 6–7 times higher than the size of nano-powder (primary size) calculated from XRD and TEM. Higher hydrodynamic size of BT NPs was due to the tendency of NPs to agglomerate in aqueous medium [19]. Zeta potential results indicated that BT NPs suspended in water had positive surface charges, whereas BT NPs suspended in culture medium had negative surface charges. Negative surface charges of BT NPs in culture media could be due to adsorption of negatively charged proteins on the surface of NPs. Physicochemical characterization of ZnO NPs (positive control) in distilled water and culture medium is also given in Table 1 [25].

### 3.2. Cytotoxic Response of BaTiO_3_ Nanoparticles

**Figure 3:**
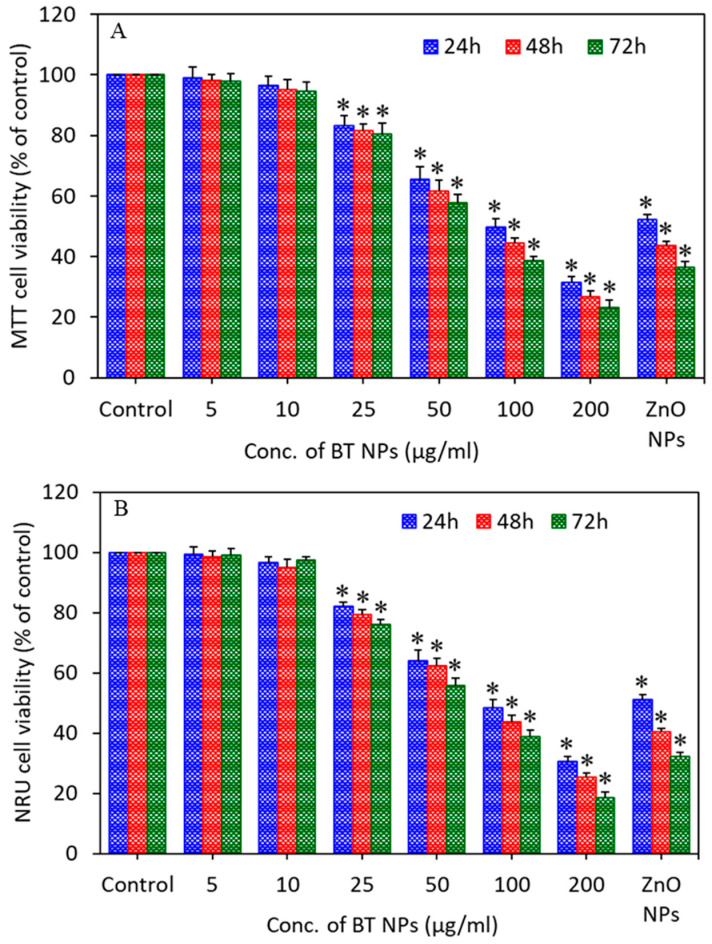
Cell viability of A549 cells exposed for 24, 48, and 72 h to different concentrations (5–200 µg/mL) of BT NPs. (**A**) MTT cell viability. (**B**) NRU cell viability. ZnO NPs (25 µg/mL for 24 h) were used as a positive control. Data are provided as mean ± SD of three independent experiments (*n* = 3). * Indicates statistically significant difference from the control group (*p* < 0.05).

Cells were exposed to different concentrations (5–200 µg/mL) of BT NPs for different time intervals (24, 48, and 72 h), and cell viability was measured by MTT and NRU assays. The MTT assay is utilized to determine metabolic activity as an indicator of cell viability. The NRU assay is based on the capability of live cells to integrate and bind the supravital dye neutral red (NR) in the lysosomes. MTT results showed that BT NPs decreased cell viability in a concentration (25–200 µg/mL)- and time (24–72 h)-dependent manner (Figure 3A). In agreement with MTT data, NRU results also demonstrated that BT NPs decreased cell viability in a concentration (25–200 µg/mL)- and time (24–72 h)-dependent manner (Figure 3B). Moreover, the positive control ZnO NPs (25 µg/mL) also decreased the cell viability time-dependently. A recent study also reported that nitrogen-doped TiO_2_ NPs inhibit the proliferation of malignant MDA-MB-231 breast cancer epithelial cells [39]. Based on cell viability results, we utilized the 25–100 µg/mL concentration range for further apoptosis and oxidative stress studies. 

**Figure 4:**
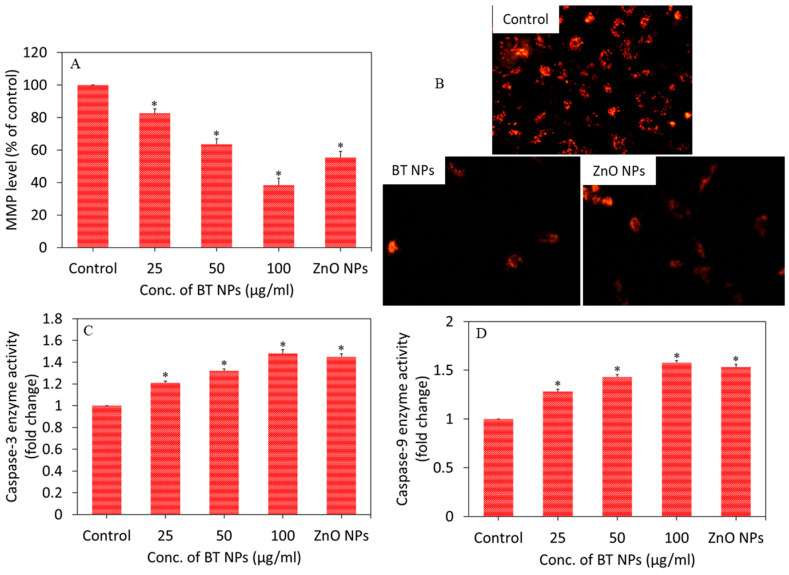
(**A**) MMP level of A549 cells exposed for 24 h to different concentrations (25–100 µg/mL) of BT NPs. (**B**) Fluorescent images of the Rh-123 probe (indicator of MMP level) after exposure to BT NPs (50 µg/mL) for 24 h. (**C** and **D**) Caspase-3 and caspase-9 enzyme activity of A549 cells exposed for 24 h to different concentrations (25–100 µg/mL) of BT NPs. ZnO NPs (25 µg/mL for 24 h) was used as a positive control. Data are provided as mean ± SD of three independent experiments (*n* = 3). * Indicates statistically significant difference from the control group (*p* < 0.05).

Apoptotic response of BT NPs in A549 cells was examined by measuring the MMP level and activity of caspase-3 and -9 enzymes. Apoptosis is triggered by various factors such as stress, nutrient deficiency, starvation, foreign particles, and certain drugs [40]. MMP is a reliable indicator of cellular stress and ongoing processes of apoptosis. Our quantitative analysis suggested that BT NPs induced MMP loss in a dose-dependent manner (25–100 µg/mL) (Figure 4A). Fluorescent microscopy data also depicted that the brightness of the Rh-123 probe (red fluorescence) was lower in BT NP-treated cells in comparison to controls (Figure 4B). The brightness of the red fluorescence of the Rh-123 probe decreased with decreasing MMP level. ZnO NPs also decreased the MMP level of A549 cells. Caspases belongs to the proteases family present in mitochondria and play crucial roles in the apoptotic pathway [41]. Among these proteases, caspase-3 and -9 are known to be critically involved in initiation and execution of the apoptotic process. Cytochrome c releases from the mitochondrial intermembrane space and binds to apoptotic protease activating factor-1 (Apaf-1) that further binds with pro-caspase-9 to form a complex called apoptosome. This complex cleaves the pro-caspase-9 to its active form, caspase-9, which further activates caspase-3. Activated caspase-3 can cleave several structural and regulatory proteins involved in the apoptotic process [42]. In the present study, we observed that BT NPs induced the activity of caspase-3 and -9 enzymes in a dose-dependent manner (Figure 4C,D). Positive control ZnO NPs also decreased the MMP level and increased the activity of caspase-3 and -9 enzymes in A549 cells. Marino and co-workers reported that remote ultrasound-mediated piezo-stimulation of BT NPs allowed in vitro growth of glioblastoma cells to be significantly decreased [4]. Another study reported that BT NPs in combination with tumor-treating fields exhibited antitumor activity against breast cancer cells by manipulating the cell cycle-related apoptosis pathway [3]. The apoptotic response of other ceramic NPs were also previously reported [9]. 

### 3.3. Oxidative Stress Response of BaTiO_3_ Nanoparticles

**Figure 5:**
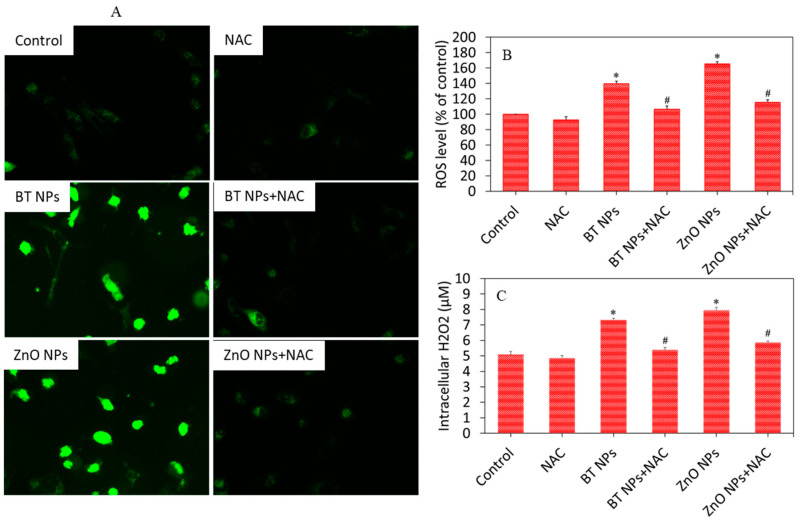
Pro-oxidant levels of A549 cells exposed for 24 h to 50 µg/mL of BT NPs with or without NAC (2 mM). ZnO NPs (25 µg/mL for 24 h) were used as a positive control. (**A**) Fluorescent microscopic images of DCF intensity in cells (an indicator of ROS generation). (**B**) Quantitative analysis of intracellular ROS level. (**C**) Quantitative analysis of intracellular H_2_O_2_ level. Data are provided as mean ± SD of three independent experiments (*n* = 3). * Indicates statistically significant difference from the control group (*p* < 0.05). ^#^ Indicates that NAC effectively abrogated the effect of BT NPs or ZnO NPs.

Studies have suggested that NPs induce cytotoxicity through oxidative stress. Oxidative stress is the state where excess pro-oxidant generation limits the antioxidant defense capacity of the cells [43]. Excessive generation of ROS leads the disturbance of cellular redox homeostasis and/or injury to cell macromolecules [44]. In this study, we assessed the ROS, H_2_O_2_, GSH, and activity of several antioxidant enzymes in A549 cells exposed for 24 h to 50 µg/mL of BT NPs in the presence or absence of NAC (ROS scavenger). Fluorescence microscopy data demonstrated that the brightness of the DCF probe (marker of ROS generation) increased in cells exposed to BT NPs or ZnO NPs as compared to control cells (Figure 5A). These images also suggested that the increased level of ROS in BT NPs or ZnO NP-treated cells was effectively abrogated by NAC co-exposure (Figure 5A). Quantitative data of ROS generation also indicated that significant increases in ROS levels due to BT NP or ZnO NP exposure were efficiently alleviated by NAC co-exposure (Figure 5B). Pro-oxidant H_2_O_2_ levels in BT NP or ZnO NP exposed cells were significantly higher as compared to controls (Figure 5C). Again, BT NP- or ZnO NP-induced H_2_O_2_ level was successfully mitigated by NAC co-exposure.

**Figure 6:**
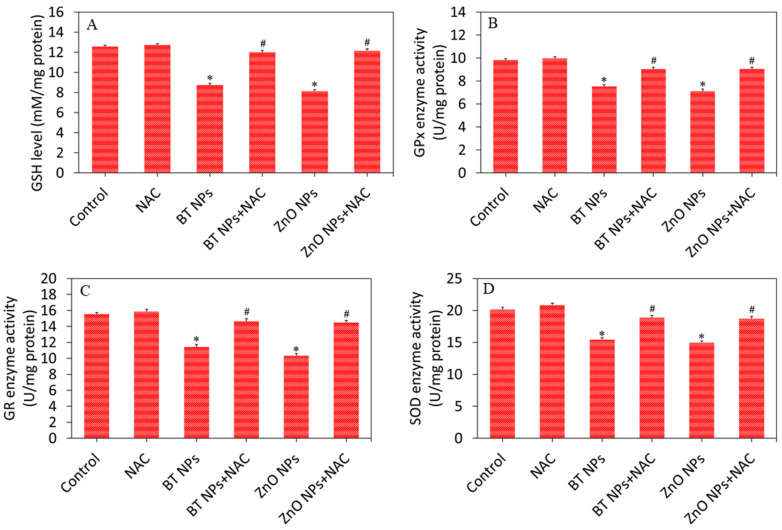
Antioxidant levels of A549 cells exposed for 24 h to 50 µg/mL of BT NPs with or without NAC (2 mM). ZnO NPs (25 µg/mL for 24 h) was used as a positive control. (**A**) GSH level. (**B**) GPx enzyme activity. (**C**) GR enzyme activity. (**D**) SOD enzyme activity. Data are provided as mean ± SD of three independent experiments (*n* = 3). * Indicates statistically significant difference from the control group (*p* < 0.05). ^#^ Indicates that NAC effectively abrogated the effect of BT NPs or ZnO NPs.

We further examined the effect of BT NPs on the antioxidant defense system of A549 cells. Cells were exposed for 24 h to 50 µg/mL of BT NPs with or without NAC. Results demonstrated that BT NPs or ZnO NPs significantly decreased the GSH level and as well as the activity of several antioxidant enzymes (GPx, GR, and SOD) in A549 cells as compared to controls (Figure 6A–D). We further noticed that the effects of BT NPs or ZnO NPs on antioxidant defense systems were successfully reverted by NAC co-exposure. These results suggested that BT NPs have potential to generate oxidative stress in A549 cells in a similar fashion as in ZnO NPs. Barium oxide (BaO) NPs also exhibited genotoxic and apoptotic effects in L929 cells, most likely due to generation of ROS [45]. An earlier study demonstrated that Ag-doped TiO_2_ NPs induced reactive oxygen species generation and oxidative stress in various types of human cancer cells [19]. 

### 3.4. BaTiO_3_ Nanoparticles Induced Cytotoxicity via Oxidative Stress

**Figure 7:**
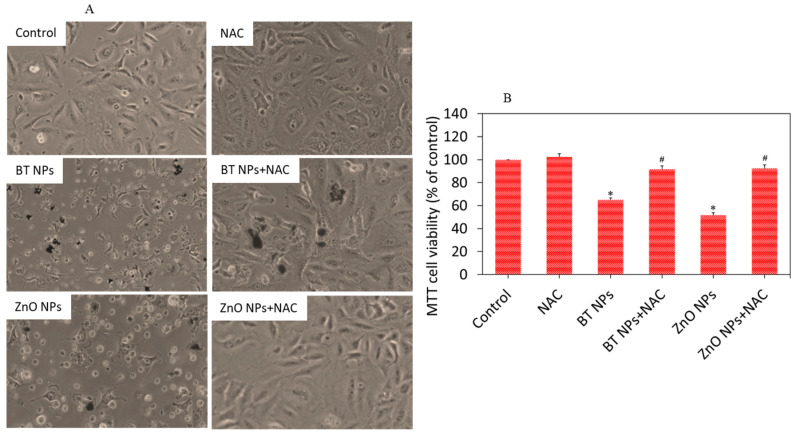
BT NP-induced cytotoxicity was mediated through oxidative stress. Cells were exposed for 24 h to 50 µg/mL of BT NPs with or without NAC (2 mM). ZnO NPs (25 µg/mL for 24 h) were used as a positive control. (**A**) Cell morphology. (**B**) MTT cell viability. * Indicates statistically significant difference from the control group (*p* < 0.05). ^#^ Indicates that NAC effectively abrogated the cytotoxic effect of BT NPs or ZnO NPs.

We examined the role of oxidative stress in BT NP-induced cytotoxicity in A549 cells. It has been reported that oxidative damage of cells due to particles or drugs can be minimized by supplementation of external antioxidants such as vitamins and NAC. For example, NAC has shown potential to mitigate the oxidative damage of cells through ROS scavenging and GSH replenishment [46]. Our data also showed that cytotoxicity exerted by BT NPs or ZnO NPs was effectively attenuated by NAC co-exposure. As we can see in Figure 7A, cell damage induced by BT NPs was significantly reverted by NAC co-exposure. MTT data (Figure 7B) also demonstrated that reduction in cell viability after exposure to BT NPs was successfully reverted when co-exposed with NAC. These results suggested that BT NP-induced cytotoxicity was mediated through oxidative stress. Most importantly, the cytotoxicity mechanism of BT NPs was similar in mode of action to that of ZnO NPs. Copper doping also enhances oxidative stress-mediated-cytotoxicity of TiO_2_ NPs in A549 cells [47]. Our previous study also showed that Zn-doped TiO_2_ induced cytotoxicity in human breast cancer (MCF-7) via oxidative stress [23]. Elucidating the possible mechanisms of selective toxicity of BT NPs against cancer cells still remains a daunting task.

It cannot be dismissed that NPs, particularly when they were not stored under sterile condition, may be contaminated with endotoxins during preparation or handling. Therefore, endotoxin contamination might influence the toxicity of NPs [48]. Endpoint chromogenic limulus amebocyte lysate (LAL) assay further confirmed that prepared BT NPs were not contaminated from endotoxins (Figure S1 of Supplementary Materials).

### 3.5. Effect of BaTiO_3_ Nanoparticles on Non-Cancerous Normal Cells

**Figure 8:**
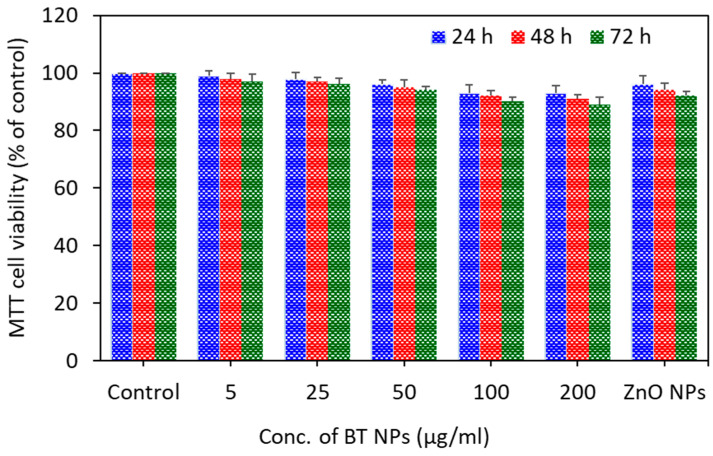
Effect of BaTiO_3_ NPs in non-cancerous human lung fibroblasts (IMR-90). Cells were exposed for 24, 48, and 72 h to different concentrations (5–200 µg/mL) of BT NPs, and cytotoxicity was determined by MTT assay. ZnO NPs (25 µg/mL) was used as a positive control. Data are provided as mean ± SD of three independent experiments (*n* = 3).

To see the selective toxicity of BT NPs, we assessed the effects of BT NPs in non-cancerous human lung fibroblasts (IMR-90). Results showed that BT NPs did not exert toxicity to IMR-90 cells (Figure 8). As expected, ZnO NPs also did not induce cytotoxicity to IMR-90 cells. The benign nature of ZnO NPs was reported previously [20,21]. Dubey and co-workers found that BT NPs did not induce any systemic toxicity in BALB/c mice [49]. Selective toxicity of semiconductor NPs against cancer cells is now being investigated by researchers [50,51].

## 4. Conclusions

We observed that BT NPs induce a dose- and time-dependent cytotoxicity to human lung carcinoma (A549) cells. MMP loss and higher activity of caspase-3 and -9 enzymes due to BT NP exposure were also observed. Moreover, BT NP-induced cytotoxicity, pro-oxidant generation, and antioxidant depletion were effectively attenuated by co-exposure of an antioxidant (N-acetyl-cysteine), suggesting that BT NP-induced toxicity was mediated through oxidative stress. Importantly, cytotoxicity mechanisms of BT NPs against human lung cancer cells were similar in mode of action to those of ZnO NPs. Overall, this preliminary study indicated that BT NPs have potential to kill cancer cells while sparing their normal counterparts. This preliminary report warrants further research on cytotoxicity mechanisms of BT NPs against various types of cancer cells and their normal counterparts.

## Figures and Tables

**Table 1:** Physicochemical characterization of BaTiO_3_ NPs and ZnO NPs.

BaTiO_3_ NPs		ZnO NPs [25]	
Parameters	Mean Value	Parameters	Mean Value
Primary size		Primary size	
TEM	16 nm	TEM	41 nm
XRD	15 nm	XRD	43 nm
Hydrodynamic size ^#^		Hydrodynamic size ^#^	
Distilled water	105 ± 5 nm	Distilled water	113 ± 7 nm
Culture medium	114 ± 3 nm	Culture medium	155 ± 6 nm
Zeta potential ^#^		Zeta potential ^#^	
Distilled water	23 ± 2 eV	Distilled water	17 ± 3 eV
Culture medium	−27 ± 3 eV	Culture medium	−19 ± 2 eV

TEM: transmission electron microscopy, XRD: X-ray diffraction, culture medium: DMEM + 10% fetal bovine serum, ^#^ data presented are mean ± SD of three independent experiments (*n* = 3).

## Supplementary Materials

The following are available online at https://www.mdpi.com/2079-4991/10/11/2309/s1.
