# Supplementary material for: Barium Titanate (BaTiO3) Nanoparticles Exert Cytotoxicity through Oxidative Stress in Human Lung Carcinoma (A549) Cells
Source: Nanomaterials (Basel). 2020 Nov 22;10(11):2309. doi: 10.3390/nano10112309 (PMC7700150; doi:10.3390/nano10112309)
Supplement: Supplementary file 1 [file nanomaterials-10-02309-s001.pdf]

Supplementary Information

Barium titanate ($BaTiO_3$) nanoparticles exert cytotoxicity through oxidative stress in human lung carcinoma (A549) cells

Maqusood Ahamed [1, *], Mohd Javed Akhtar [1], M.A. Majeed Khan[1], Hisham A. Alhadlaq [1,2], Aws Alshamsan [3]

[1] King Abdullah Institute for Nanotechnology, King Saud University, Riyadh-11451, Saudi Arabia

[2] Department of Physics and Astronomy, College of Science, King Saud University, Riyadh-11451, Saudi Arabia

[3] Department of Pharmaceutics, College of Pharmacy, King Saud University, Riyadh-11451, Saudi Arabia

* Correspondence:

Maqusood Ahamed, Ph.D.

Associate Professor

King Abdullah Institute for Nanotechnology

King Saud University

Tel.: +966114698781

Email: mahamed@ksu.edu.sa

S1. Materials and methods

S1.1. Endpoint chromogenic limulus amebocyte lysate (LAL) assay

The endpoint chromogenic limulus amebocyte lysate (LAL) assay kit (Lonza, Basel, Switzerland) was used to examine endotoxin contamination in BT NPs. This assay has a sensitivity range of 0.1 EU/ml - 1.0 EU/ml. Briefly, BT NPs at a concentration of 50 µg/ml was were mixed with the LAL supplied in the test kit and incubated at 37 °C for 10 min. A peptide substrate solution was then mixed with the LAL-sample mixture and incubated at 37 °C for next 6 min. The reaction was then stopped by addition of stop reagent supplied with the kit. In addition to the complete reaction mixture (i.e. BT NPs+LAL+substrate), two additional mixtures were prepared to check the possible interference of BT NPs in the assay, namely BT NPs+LAL and BT NPs+substrate. If endotoxin is present in the sample, a yellow color should develop only in the complete reaction mixture, not in other two mixtures. The absorbance of the enzymatically cleaved p-nitroaniline part of the substrate peptide was measured at 405 nm in a microplate reader (Synergy-HT, BioTek, Vinnoski, VT, USA). Since this absorbance is in direct proportion to the amount of endotoxin present, the concentration of endotoxin can be calculated from a standard curve using LPS.

S1.2. MTT assay

Cell viability was measured by MTT assay [1] with some specific changes [2]. This assay measures the mitochondrial function by determining the ability of living cells to reduce MTT into blue formazon product. Briefly, 20,000 cells/well seeded in a 96-well plate and allowed for 24 h to attach on the surface of the plate. Then, cells were treated to different concentrations of BT NPs (0-200 µg/ml) for different exposure

times (24, 48, and 72 h). After the completion of exposure time, culture medium was removed from each well to avoid interference of BT NPs and replaced with new medium containing MTT solution in an amount equal to 10% of culture volume. The 96-well plate is now incubated for 3 h at 37 °C until a purple colour formazan product was appeared. The resulting formazan product was dissolved in acidified isopropanol. Further, 96-well plate was centrifuged at 2300×g for 5 min to settle down the BT NPs, if present in the solution. Then, a 100 μl supernatant was transferred to other fresh wells of 96-well plate and absorbance was measured at 570 nm using a microplate reader (Synergy-HT, BioTek, Vinnoski, VT, USA).

S1.3. NRU assay

Neutral red uptake (NRU) assay was performed following the procedure as described by Borenfreund and Puerner [3] with some specific changes [2]. Briefly, 20,000 cells/well were seeded in 96-well plates and allowed for 24 h to attach on the surface of the plate. Then, cells were treated to different concentrations of BT NPs (0-200 μg/ml) for 24, 48, and 72 h time intervals. After the completion of exposure time, culture medium was aspirated and cells were washed with phosphate buffer saline (PBS) before being incubated for 3 h in culture medium supplemented with neutral red (50 μg/ml). Then, medium was washed off rapidly with a solution containing 0.5% formaldehyde and 1% calcium chloride. Cells were further incubated for 20 min at 37 °C in a mixture of acetic acid (1%) and ethanol (50%) to extract the dye. The 96-well plates were then centrifuged at 2300×g for 5 min to settle the remaining NPs, if present in the solution. After this, 100 μl of supernatant was transferred to new wells of 96-well plate and the absorbance was recorded at 540 nm using a microplate reader (Synergy-HT, BioTek).

S1.4. Mitochondrial membrane potential assay

Mitochondrial membrane potential (MMP) was quantified using rhodamine-123 probe (Rh-123, Sigma-Aldrich) [4]. In brief, 20,000 cells/well seeded in a 96-well

plate and allowed for 24 h to attach on the surface. Then, cells were treated to different concentrations of BT NPs (25-100 μg/ml) for 24 h. ZnO NPs (25 μg/ml for 24 h) was used as a positive control. At the end of exposure, cells were harvested and washed twice with PBS. Cells were further exposed with 10 μg/ml of Rh-123 probe for 1 h at 37 °C in the dark. Cells were washed with PBS and fluorescent intensity of Rh-123 was measured at a microplate reader (Synergy-HT, BioTek). A parallel set of experiment in 24-well plate (1×10^5 cells/well) was also prepared to capture the fluorescent intensity of Rh-123 probe in control, BT NPs (50 μg/ml for 24 h) or ZnO NPs (25 μg/ml for 24 h) treated groups using a DMi8 fluorescent microscope (Leica Microsystems, GmbH, Wetzlar, Germany). Cell images were captured at 20× magnification.

S1.5. Reactive oxygen species generation assay

Intracellular ROS generation was assayed using 2,7-dichlorofluorescin diacetate (DCFH-DA) as reported in our previous work [4]. ROS level was estimated through two distinc methods; quantitative analysis and microscopic fluorescence imaging. For quantitative assay, 20,000 cells/well were seeded in a 96-well black-bottomed plates and allowed to adhere for 24 h in a CO_2 incubator at 37 °C. Cells were further exposed to different concentrations of BT NPs (25-100 μg/ml) for 24 h. ZnO NPs (25 μg/ml for 24 h) was used as a positive control. In some set of experiments, cells were co-exposed with 2 mM of N-acetylcycteine (NAC) (Sigma-Aldrich, St. Louis, MO, USA) in the presence or absence of BT NPs or ZnO NPs. Cells without NPs exposure served as control in each experiment. After the completion of exposure time, cells were washed twice with PBS before being incubated in 1 ml of working solution of DCFH-DA at 37 °C for 30 min. Then, cells were lysed in alkaline solution and centrifuged at 2300×g for 15 min to settle down the cell debris. A 100 μl supernatant was transferred to a new 96-well plate, and fluorescence was measured at 485 nm excitation and 520 nm emission using a microplate reader (Synergy-HT, BioTek). The values were expressed as a percent of fluorescence intensity relative to the control cells. A parallel set of cells in a transparent 24-well plate (1×10^5 cells/well) was

analysed for intracellular fluorescence imaging of ROS level using a DMi8 fluorescent microscope (Leica Microsystems, Leica Microsystems, GmbH, Germany). Cell images were captured at 20× magnification.

S1.6. Preparation of cell extract

In brief, cells were cultured in 6-well plate ($1×10^6$ cells/well) and exposed to different concentrations of BT NPs (25-100 μg/ml) for 24 h. ZnO NPs (25 μg/ml for 24 h) was used as a positive control. In some set of experiments, cells were co-exposed with 2 mM of NAC with or without BT NPs or ZnO NPs. After the completion of exposure time, cells were harvested in ice cold PBS by scraping and washed with PBS at 4 °C. Cell pellets were further lysed in cell lysis buffer [1X 20 mM Tris-HCl (pH 7.5), 150 mM NaCl, 1 mM Na_2EDTA, 1% Triton, 2.5 mM sodium pyrophosphate]. Following centrifugation (15000 g for 10 min at 4 °C) the supernatant (cell extract) was immediately stored at 4 °C. Cell extract was used for the measurements of caspase-3 and -9 enzymes activity, glutathione (GSH) level, and activity of several antioxidant enzymes such as glutathione peroxidase (GPx), glutathione reductase (GR), and superoxide dismutase (SOD).

S1.7. Caspase-3 and caspase-9 enzymes assay

Caspase-3 and caspase-9 enzymes activity was measured using BioVision colorimetric kits (Milpitas, CA, USA). This assay is based on the principle that activated caspases in apoptotic cells cleave the synthetic substrates to release free chromophore p-nitroanilide (pNA), which was recorded at 405 nm. The pNA produced after specific action of caspase-3 and caspase-9 on tertrapeptide substrates were DEVD-pNA and LEHD-pNA, respectively. In brief, reaction mixture consisted of 50 μl of control and treated cell extract protein (50 μg), 50 μl of 2X reaction buffer (containing 10 mM dithiothreitol) and 5 μl of 4 mM DEVD-pNA (for caspase- 3) or LEHD-pNA (for caspase-9) substrate in a total volume of 105 μl. The reaction mixture

was incubated at 37 °C for1 h and absorbance of the product was recorded at 405 nm by a microplate reader (Synergy-HT, BioTek).

S1.8. Glutathione assay

The glutathione (GSH) level was quantified using Ellman's method [5]. Briefly, a mixture of 0.1 ml of cell extract and 0.9 ml of 5% TCA was centrifuged (2500×g) for 15 min at 4 °C. Then, 0.5 ml of the supernatant was added into 1.5 ml of 0.01% 5,5'-dithiobis-(2-nitrobenzoic acid (DTNB) and the reaction was monitored at 412 nm. The amount of GSH was expressed in terms of nanomole/mg protein.

S1.9. Glutathione peroxidase enzyme assay

Activity of glutathione peroxidase (GPx) enzyme was measured using protocol of Rotruck and co-workers [6]. Briefly, a reaction mixture contained 20 µl of 0.1 M GSH, 10 µl of cell extract, 100 µl of 2 mM NADPH, 100 µl of 10 U/ml glutathione reductase, 800 µl of 0.2 M Tris-HCl, and 10 µl of 5 mM t-butylhydroperoxide. The oxidation rate of NADPH was monitored at 320 nm.

S1.10. Glutathione reductase enzyme assay

Glutathione reductase (GR) enzyme activity was measured using method of Carlberg and Mannervik [7]. The method is based on the oxidation of NADPH to $NADP^+$ catalysed by GR. Activity of GR was estimated by following reduction in absorbance at 340 nm due to oxidation of NADPH. The reaction mixture contained 25 mM phosphate buffer (pH 7.8), 0.5 mM oxidized glutathione, 0.12 mM NADPH and an aliquot of the cell extract. Correction was made for oxidation of NADPH in the absence of oxidized glutathione.

S2. Results

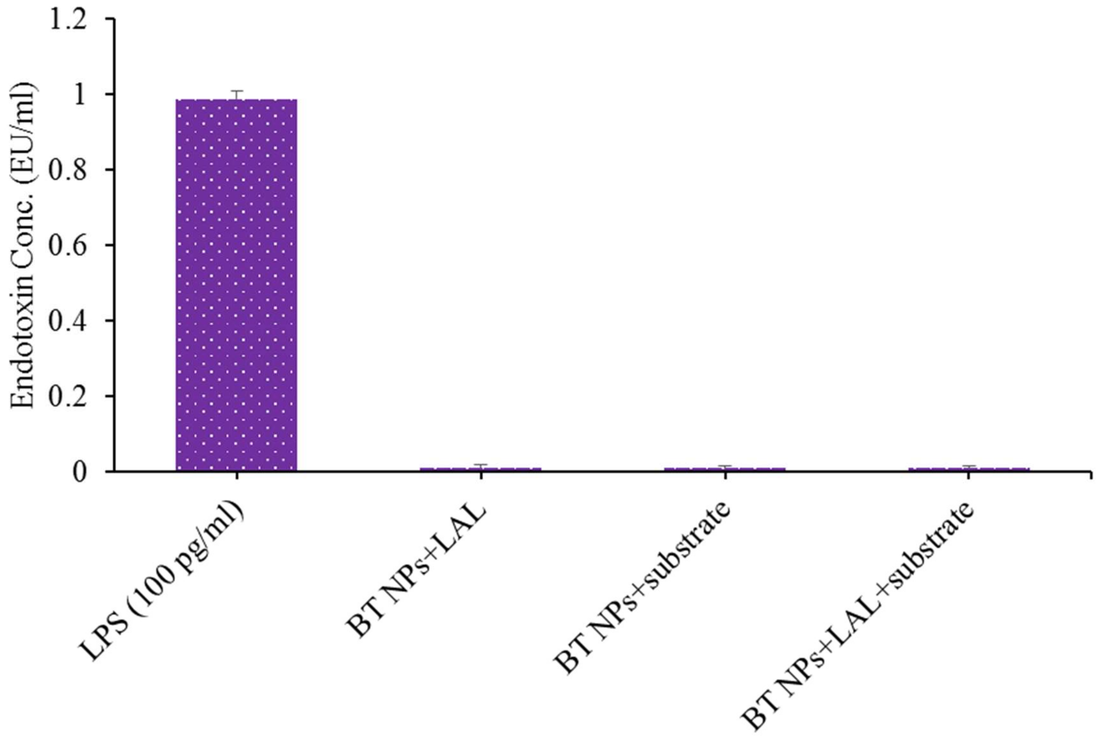

Figure S1. Endpoint chromogenic limulus amebocyte lysate (LAL) assay for endotoxin detection in BT NPs. A concentration of 50 μg/ml of BT NPs were incubated with LAL (containing enzyme), or substrate, or both (LAL+substrate). After the completion of incubation time, absorbance of the substrate was measured at 405 nm. If endotoxin is present in the sample, a yellow color should develop only in the complete reaction mixture (BT NPs+LAL+substrate), not in other two mixtures. The absorbance of the enzymatically cleaved p-nitroaniline part of the substrate peptide was measured at 405 nm by a microplate reader (Synergy-HT). Results showed that there is no endotoxin contamination in prepared BT NPs.

References

1. T. Mosmann, Rapid colorimetric assay for cellular growth and survival: Application to proliferation and cytotoxicity assays, Journal of Immunological Methods. 65 (1983) 55–63. https://doi.org/10.1016/0022-1759(83)90303-4.
2. M. Ahamed, M.J. Akhtar, M.A. Siddiqui, J. Ahmad, J. Musarrat, A.A. Al-Khedhairy, M.S. AlSalhi, S.A. Alrokayan, Oxidative stress mediated apoptosis

induced by nickel ferrite nanoparticles in cultured A549 cells, Toxicology. 283 (2011) 101–108. https://doi.org/10.1016/j.tox.2011.02.010.

3. Borenfreund, E. & Puerner, J. A. A simple quantitative procedure using monolayer culture for toxicity assays. J. Tissue Cult. Methods 9, 7–9 (1984).
4. M.A. Siddiqui, H.A. Alhadlaq, J. Ahmad, A.A. Al-Khedhairy, J. Musarrat, M. Ahamed, Copper Oxide Nanoparticles Induced Mitochondria Mediated Apoptosis in Human Hepatocarcinoma Cells, PLoS ONE. 8 (2013). https://doi.org/10.1371/journal.pone.0069534.
5. G.L. Ellman, Tissue sulfhydryl groups, Archives of Biochemistry and Biophysics. 82 (1959) 70–77. https://doi.org/10.1016/0003-9861(59)90090-6.
6. J.T. Rotruck, A.L. Pope, H.E. Ganther, A.B. Swanson, D.G. Hafeman, W.G. Hoekstra, Selenium: Biochemical role as a component of glatathione peroxidase, Science. 179 (1973) 588–590. https://doi.org/10.1126/science.179.4073.588.
7. I. Carlberg, B. Mannervik, [59] Glutathione reductase, Methods in Enzymology. 113 (1985) 484–490. https://doi.org/10.1016/S0076-6879(85)13062-4.
